# Hollow vaginal stent for a case of Mayer-Rokitansky-Kuster-Hauser syndrome: a case report

**DOI:** 10.11604/pamj.2021.38.343.28837

**Published:** 2021-04-09

**Authors:** Vikram Murlidhar Belkhode, Sharayu Vinod Nimonkar

**Affiliations:** 1 Department of Prosthodontics, Shard Pawar Dental College and Hospital, Datta Meghe Institute of Medical Sciences, Sawangi, Wardha, Maharashtra, India

**Keywords:** Mayer-Rokitansky-Kuster-Hauser syndrome, hollow prosthesis, vaginal agenesis, vaginal stent, case report

## Abstract

Congenital vaginal agenesis is a common condition with an instance of 1 in 5000 females. It is usually associated with Mayer-Rokitansky-Kuster Hauser syndrome. Such anomalies have a high impact on the physiology and psychology of patients. A simple approach for the hollowing of the customized vaginal stent prosthesis has been described for the case of Mayer-Rokitansky-Kuster-Hauser syndrome.

## Introduction

The Mayer-Rokitansky-Kuster-Hauser (MRKH) syndrome is a congenital anomaly identified by congenital aplasia of the uterus and upper part of the vagina, manifesting amenorrhea at adolescence. It has an autosomal dominant mode of inheritance with incomplete penetrance and variable expressivity [1]. There are several surgical and nonsurgical treatment options for treating MRKH. The results of surgical treatment of creating neovaginal cavity have been proved to be more promising than the nonsurgical [2]. The use of vaginal stent after surgery remains the cornerstone of the treatment. It maintains the patency of surgically created neovaginal cavity by prevents post-surgical contraction. Reducing the weight of vaginal stent by making it hollow is beneficial as the heavyweight may lead to perforations of underlying vital tissues and organs. This article presents a simple technique of fabricating a customized hollow acrylic vaginal stent for a young female patient with MRKH syndrome treated surgically by creating a neovagina cavity using Abbe-McIndoe technique.

## Patient and observation

An 18-year young girl reported to the department of prosthodontics with a referral from the department of surgery for vaginal stent prosthesis. Past medical history revealed that she was diagnosed with MRKH syndrome. Treatment formulated for the case was creating a neovaginal cavity by the Abbe-McIndoe technique. A hollow vaginal stent was customized with acrylic resin for this case. An informed consent was signed before starting the treatment.

**Figure 1:**
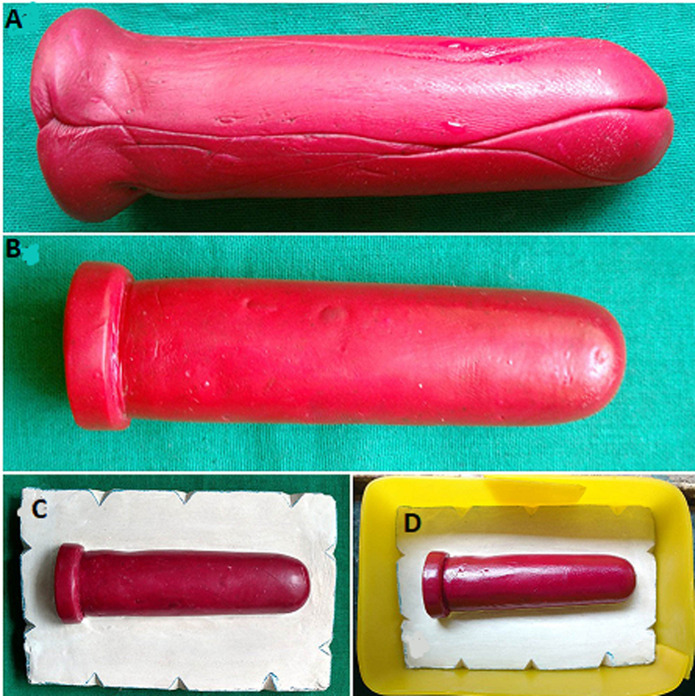
A) scaffold; B) scaffold invested in dental plaster; C) boxed base pour; D) two-piece mold with orientation groves

**Figure 2:**
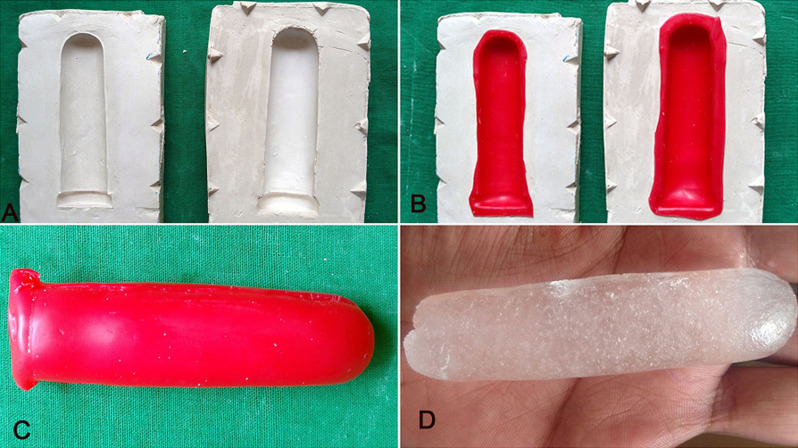
A) wax sheet adapted over the walls of the mold; B) wax pattern; C) cylindrical ice block interposed between the heat cure acrylic resin for curing

**Figure 3:**
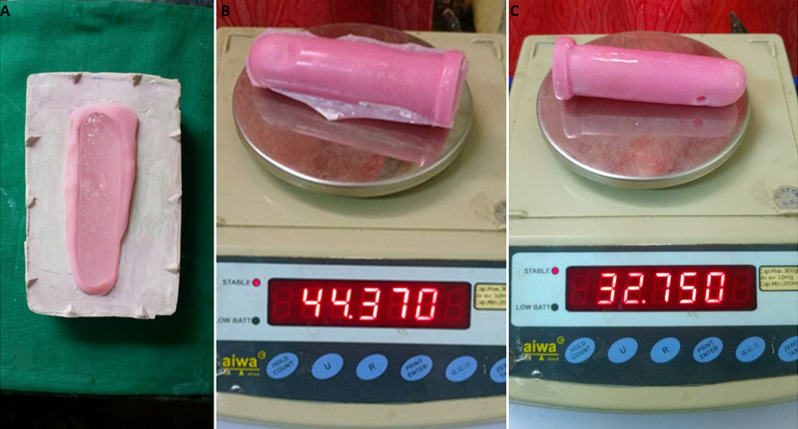
A) weight of the prosthesis after hollowing; B) weight of the prosthesis before hollowing

**Fabrication of the vaginal stent:** on the basis of the thickness of the intervening tissue between the perineum and pelvic peritoneum, examined over a magnetic resonance imaging (MRI) scan, the dimension of the neovaginal cavity was calculated to be 12cm (depth) x 5cm (Width) (Figure 1, Figure 2, Figure 3). A scaffold with this dimension was made in a modeling compound (dents). It was then invested to form a two-piece mold. Orientation groves were made on base pour for its orientation with the counter mold. The modeling wax (pyrex) was softened and contoured on the walls of both the molds and was closed to form a hollow wax pattern of the stent. Water was injected in the wax pattern with the help of a syringe and was frozen in an upright position to form a cylindrical ice block. The heat cure acrylic resin was mixed and placed in two half of the mold and the cylindrical ice block was interposed between it and was cured. After curing the stent was retrieved and a hole was drilled to remove the water. The hole was closed with the self-cure acrylic resin. The stent was finished, polished and disinfected.

**Surgical procedure:** the patient was put down in a lithotomy position and was catheterized. General anesthesia was employed and Abbe-McIndoe technique was performed to create a neovagina cavity by giving a horseshoe-shaped midline incision at the introitus. The fibroconnective tissues between the bladder and rectum were dissected. A neovaginal cavity was formed and was enlarged to 13cm in-depth and 5cm in diameter as predetermined. A split-thickness skin graft was harvested from the gluteal area of dimension 13cm x 5cm and was wrapped over the customized hollow vaginal stent. The raw surface of the graft was placed facing outwards and was inserted into the neovagina cavity created. The vaginal stent was retained in position for 7 days.

**Follow up:** after 7 days vaginal stent was removed and healing of the graft was inspected. The patient was encouraged to use the vaginal stent for 6 months post-surgically for maximum period of time even during sleep to avoid the replapse (fibrosis). Post insertion instructions regarding the use and maintenance of the vaginal stent were given. The patient´s compliance was satisfactory.

## Discussion

Genital abnormality associated with a girl child is considered to be an onerous burden to her parents. Mayer-Rokitansky-Kuster-Hauser syndrome is a condition with an instance of 1 in 5000 females. The Abbe McIndoe technique is the most recommended procedure for vaginal agensis. Advantage of this technique is its lower complication rate, trans-abdominal approach, and less surgical risk. The ultimate goal of the surgery is to create a cavity for an adequate passageway for penetration and to facilitate satisfactory sexual intercourse [3]. The surgeon opted for gluteal graft in this case as it satisfied the requirement of being a highly vascularized skin paddle with the same thickness and width needed for the construction of the neovaginal cavity. However, literature has also documented such cases successfully treated with thigh flap. Various methods, materials, and techniques have been used by different authors to fabricate the vaginal stent [4]. The ice used in this technique for hollowing was effortlessly removed and also ensured the uniform and adequate thickness of resin all around the planned hollow cavity in the prosthesis. This hollow vaginal stent can also act as a drain and a passage for intracanal medicament in this region. Moreover, the use of hard acrylic material for stent fabrication overcomes the problems associated with the fungal contamination as seen with soft silicon material [5]. The chances of developing fistula were bare minimal as the stent was highly finished and polished without any sharp edges. Proper counseling and motivation for use of vaginal stent post-operatively play a guiding role for more reliable results and the long term success of the treatment.

## Conclusion

Creating neovaginal space is a treatment of choice for the congenital vaginal agenesis cases. Combined teamwork by gynecologists, psychiatrists, and prosthodontists can give predictable successful results in such cases. Early diagnosis and primary care is must to improve the quality of life among such patients.
